# Eating less suppresses microRNA assassins in the brain

**DOI:** 10.18632/aging.100287

**Published:** 2011-02-24

**Authors:** Emmette Hutchison, Mark P. Mattson

**Affiliations:** ^1^ Laboratory of Neurosciences, National Institute on Aging Intramural Research Program, Baltimore, MD 21224, USA; ^2^ Neuroscience Graduate Program, Brown University, Providence, RI 02906, USA; ^3^ Department of Neuroscience, Johns Hopkins University School of Medicine, Baltimore, MD 21205, USA

The aging brain is perhaps the most fragile of tissues, with a population of structurally elaborate and metabolically demanding neurons that are seldom replenished during adulthood. Neurodegenerative disorders such as Alzheimer's disease (AD) and acute insults such as an ischemic stroke lead to seemingly irreparable damage that impairs cognitive and/or motor function. Considerable evidence suggests that the vulnerability of neurons to aging and associated disorders can be modified by environmental factors including dietary energy intake and exercise [1, 2, 3]. Energy (caloric) restriction (CR) and exercise induce the expression of neurotrophic factors, including brain-derived neurotrophic factor (BDNF), cytoprotective protein chaperones, and proteins that can prevent apoptosis. The mechanisms responsible for the neuro-protective effects of CR and exercise are mediated, in part, by changes in gene transcription [3]. However, new findings reported in this issue of Aging suggest a role for down-regulation of microRNAs (miRNAs) that target mRNAs encoding cell survival proteins in the beneficial effects of CR on the aging brain [4].

miRNAs are short non-coding RNAs that typically bind to scores of transcripts an inhibit translation of the targeted mRNA [5]. Khanna et al found that levels of three miRNAs (miR-34a, miR30e and miR181-a-1*) are significantly lower in brain tissue samples from old mice (24-28 months-old) that had been maintained on a CR diet (40% CR beginning at 4 months of age) compared to mice on the usual ad libitum diet. Interestingly, all three of these miRNAs were predicted to have at least one target site for Bcl2, an anti-apoptotic protein previously been shown to increase with CR. The authors confirmed the repressive action of these miRNAs on Bcl2. These changes in miRNA expression were validated in both cortical and hippocampal tissues, suggesting a more global repression of these miRNAs in the CNS due to CR.

Although the authors chose to solely investigate Bcl2 as a target of these three miRNAs in mediating the beneficial effects of CR, eight additional shared mRNA targets were found using multiple target-prediction algorithms. One of these targets, cAMP response element binding protein 1 (CREB1), is an important activator of several immediate response genes that are critical to synaptic plasticity [6]. Repression of CREB1 by these three age-dependent miRNAs could play a role in the cognitive decline observed with aging. Additionally, huntingtin (Htt) is a predicted target of these miRNAs, and this possible interaction may play a role in the beneficial effects of dietary energy restriction in Htt mutant mice, a model of Huntington's disease [7]. Moreover, previous research demonstrated that miR-30a, which has an identical seed region to miR-30e acts to functionally repress BDNF expression in the cortex [8]. Up-regulation of BDNF by CR has been shown to mediate, in part, the increased neurogenesis by CR and is also thought to play an important role in learning and memory [9, 10]. Although not explored by Khanna and colleagues in the context of CR, the regulation of BDNF by the miR-34 family represents yet another potential avenue for miRNAs as mediators of effects of dietary energy intake on neuronal vulnerability in aging and disease.

It will be important to determine whether changes in the expression of miRNAs 34a, 30e, and 181a do in fact mediate effects of energy intake on neuronal vulnerability. This might be accomplished by overexpressing or knocking down each of these miRNAs in neurons of interest in animal models of Alzheimer's, Parkinson's and Huntington's diseases. Whether Bcl-2 is a pivotal target of this miRNAs could be determined in experiments with Bcl-2 deficient mice. It seems likely that the findings of Khanna et al. [4] represent the ‘tip of the iceberg’ with regards to miRNAs in brain aging and disease as there are undoubtedly numerous other miRNAs and target mRNAs involved in regulating neuronal survival and plasticity. This arena of research is a promising area for the development of novel therapeutic interventions in neurodegenerative disorders. Indeed, multiple miRNAs appear to be dysregulated in neurological diseases [5]. Initial studies in non-human primates have further emphasized the potential for miRNA-based theraputics [11]. The work by Khanna and colleagues suggest a potential for miRNA-based therapies that utilize the same anti-apoptic mechanisms as CR. However, as a single miRNA can regulate hundreds of transcripts, systemic delivery of a miRNA mimetic or sponge may result in undesirable off-target and tissue specific effects.
